# An extensive visual data for reliable identification of medicinal plant leaves

**DOI:** 10.1016/j.dib.2025.112129

**Published:** 2025-10-10

**Authors:** Md. Mafiul Hasan Matin, Md. Sefatullah, Md Shariar Kabir, Haifa Binte Habib

**Affiliations:** a Department of Computer Science and Engineering, Netrokona University, Netrokona 2400, Bangladesh; b Department of Computer Science and Engineering, Daffodil International University, Bangladesh

**Keywords:** Medicinal plant dataset, Image recognition, Computer vision, Deep learning, Image classification, Medicinal leaves dataset

## Abstract

This study presents a high-quality, labeled image dataset of medicinal plant leaves designed to support machine vision and computer vision research. The dataset comprises 1380 original RGB images spanning six medicinal plant species Arjun Leaf, Curry Leaf, Marsh Pennywort Leaf, Mint Leaf, Neem Leaf, and Kalanchoe pinnata (Rubble Leaf) captured under natural lighting conditions using the iPhone 13 Pro. To enhance dataset diversity and improve model generalization, seven augmentation techniques were applied, including brightness adjustment, geometric transformations, and horizontal flipping, resulting in 9660 augmented images and a total of 11,040 samples. All images were pre-processed and resized to 512 × 512 pixels. The dataset was organized into well-structured directories and published openly via the Mendeley Data repository. This dataset offers a valuable resource for researchers developing automated medicinal plant identification systems and contributes to broader efforts in ethnobotany, agriculture, and digital health.

Specifications Table

**Table:** 

Subject	Computer Sciences
Specific subject area	Deep Learning, Computer Vision, Plant Science, Medicinal Plant, Image Classification
Type of data	Image (.jpg)
Data collection	Images were collected from nurseries after identifying medicinal plants through expert consultation. Data were captured under natural lighting using iPhone 13 Pro cameras, followed by data augmentation.
Data source location	Institution: Diploma Krishibid Nursery and Hossain Nursery Zone: Dattapara, Birulia,Savar Country: Bangladesh Latitude and Longitude: 23° 45′ 17.15″ N, 90° 22′ 34.12″ E
Data accessibility	Repository name: Mendeley Data Data identification number: 10.17632/fj93rrfv2y.2 Direct URL to data: https://data.mendeley.com/datasets/fj93rrfv2y/2
Related research article	None

## 1 Value of the Data

• The dataset contains 11,040 high-resolution RGB images of six medicinal plant leaf types, captured in standardized conditions for use in machine vision applications [[1], [2], [3]].
• It supports training and evaluation of deep learning models for multi-class image classification [4] in botanical and pharmaceutical research.
• Enables automated medicinal plant recognition [5], minimizing reliance on manual identification and supporting plant-based drug discovery and conservation.
• The structure and balance of the dataset allow for reproducible experiments and robustness testing in transfer learning and data augmentation.
• The dataset is well-suited for academic projects, theses, and applied research in computer vision, ethnobotany, and sustainable agriculture.

## 2 Background

Manual identification of medicinal plants is a time-consuming task requiring botanical expertise. As interest grows in automating plant classification using computer vision, the need for diverse, labeled image datasets becomes critical. To address this, we created a high-quality dataset of six commonly used medicinal plants through real-world image capture in local nurseries [4]. The goal was to facilitate the development of reliable deep learning models for plant classification and to contribute foundational data for future AI-based ethnopharmacological research [[7], [8], [9], [10], [11], [12], [13]].

## 3 Data Description

The dataset is structured in two compressed folders: Original Images (Version 02).zip and Augmented Images (Version 02).zip. Each folder contains six subfolders representing the six medicinal leaf classes: Arjun Leaf, Curry Leaf, Marsh Pennywort Leaf, Mint Leaf, Neem Leaf, and Kalanchoe pinnata (Rubble Leaf) shown in Fig. 3. All images are in .jpg format with a resolution of 512×512 pixels in RGB color.

**Fig. 1:**
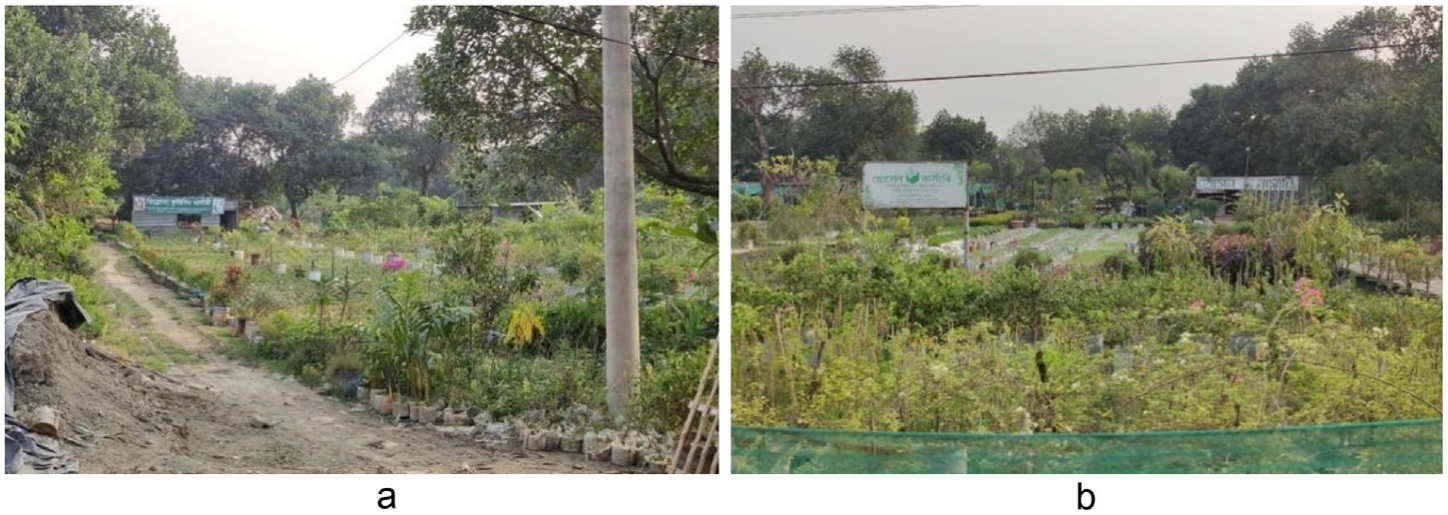
Medicinal plant nurseries (sources of the dataset).

Original images were captured using the iPhone 13 Pro camera system in natural lighting conditions with assistance from nursery staff. Fig. 1 illustrates the source environments-(a) Diploma Krishibid Nursery and (b) Hossain Nursery-located in Dattapara, Birulia, Savar, Dhaka.

**Fig. 2:**
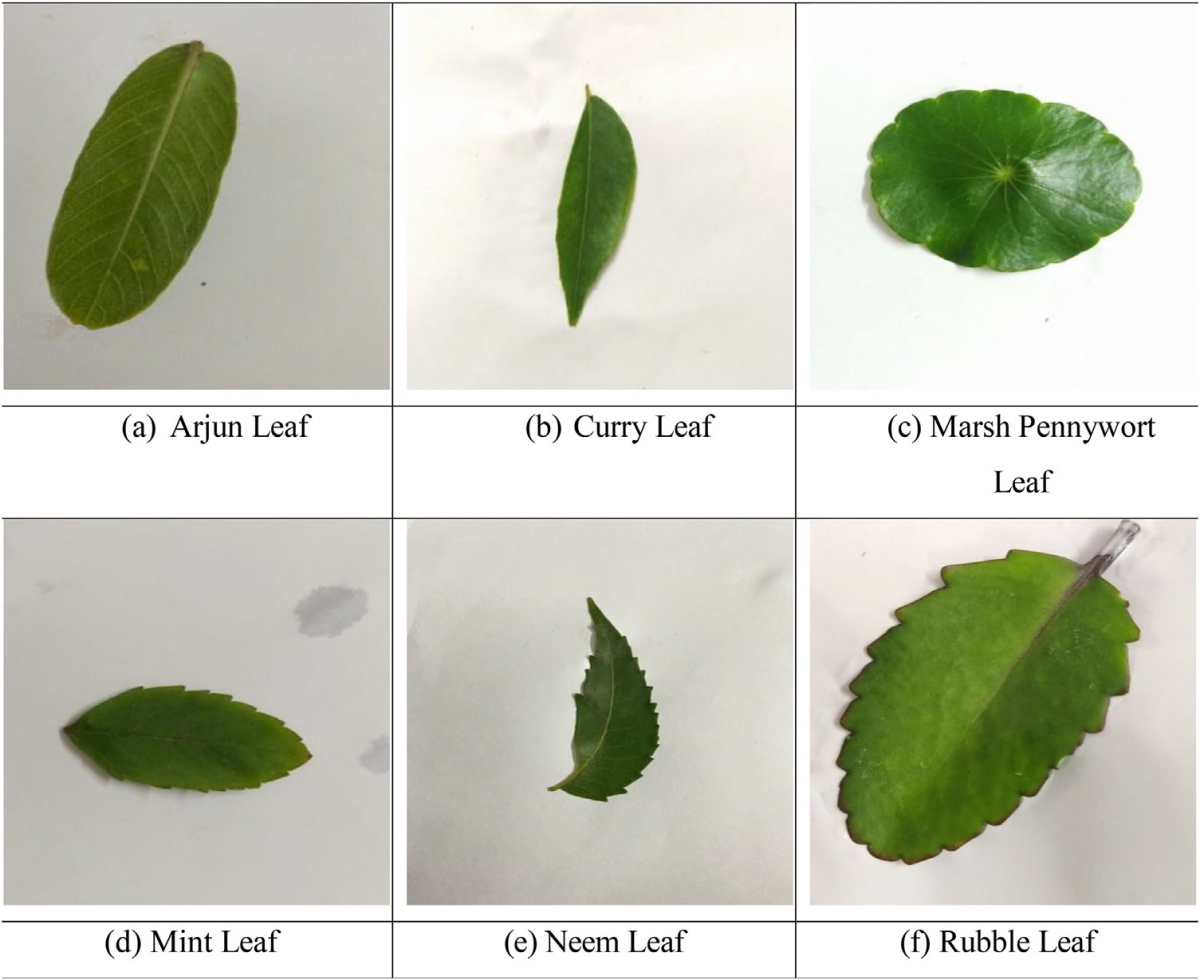
The six categories of image samples included in the medicinal plant leaf dataset. Top row: (a) Arjun Leaf (*Terminalia arjuna*) - Conical-oblong leaves with green tops and brown undersides; widely used for cardiovascular treatments, (b) Curry Leaf (Murraya koenigii) -Aromatic, bi-pinnate leaves rich in vitamins and minerals; used for managing nausea, diabetes, and hair health, (c) Marsh Pennywort (Hydrocotyle vulgaris) - Rounded leaves used for treating ulcers and urinary tract infections. Bottom row: (d) Mint Leaf (Mentha spp.) - Serrated, fragrant leaves used in teas and personal care; provides cooling and digestive relief, (e) Neem Leaf (Azadirachta indica) - Compound serrated leaves with medicinal use across infections, skin, and dental issues, (f) Kalanchoe pinnata (Rubble Leaf) (Kalanchoe pinnata) - Known for asexual propagation; used in traditional medicine for kidney stones, ulcers, and wound care.

To increase the dataset’s size and variability, we applied augmentation techniques such as histogram-equalized brightness, vertical and horizontal shifting (0.2), rotation (45°), shearing (0.2), zooming (0.2), and horizontal flipping. This resulted in 9660 augmented images, bringing the total dataset size to 11,040 images. Fig 2

**Fig. 3:**
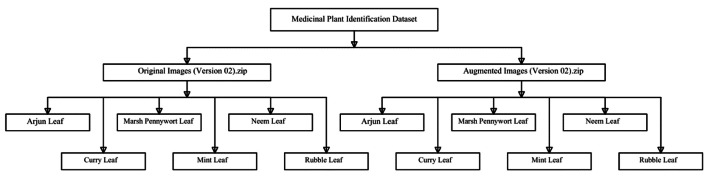
Dataset folder structure.

**Fig. 4:**
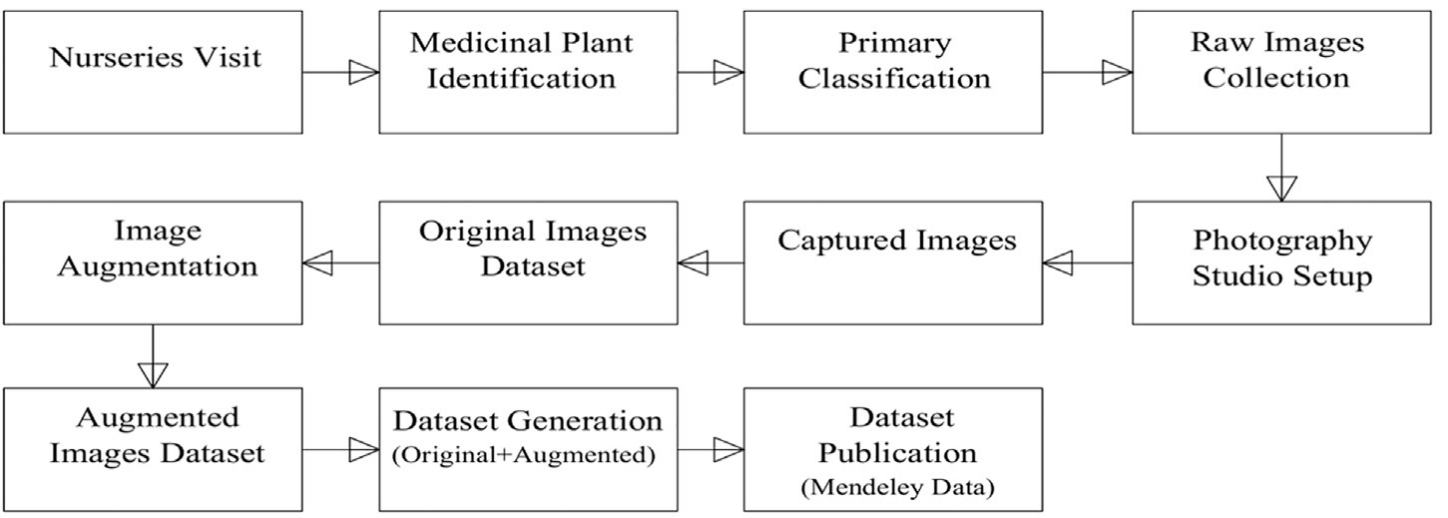
Workflow for the medicinal plant image dataset generation.

**Fig. 5:**
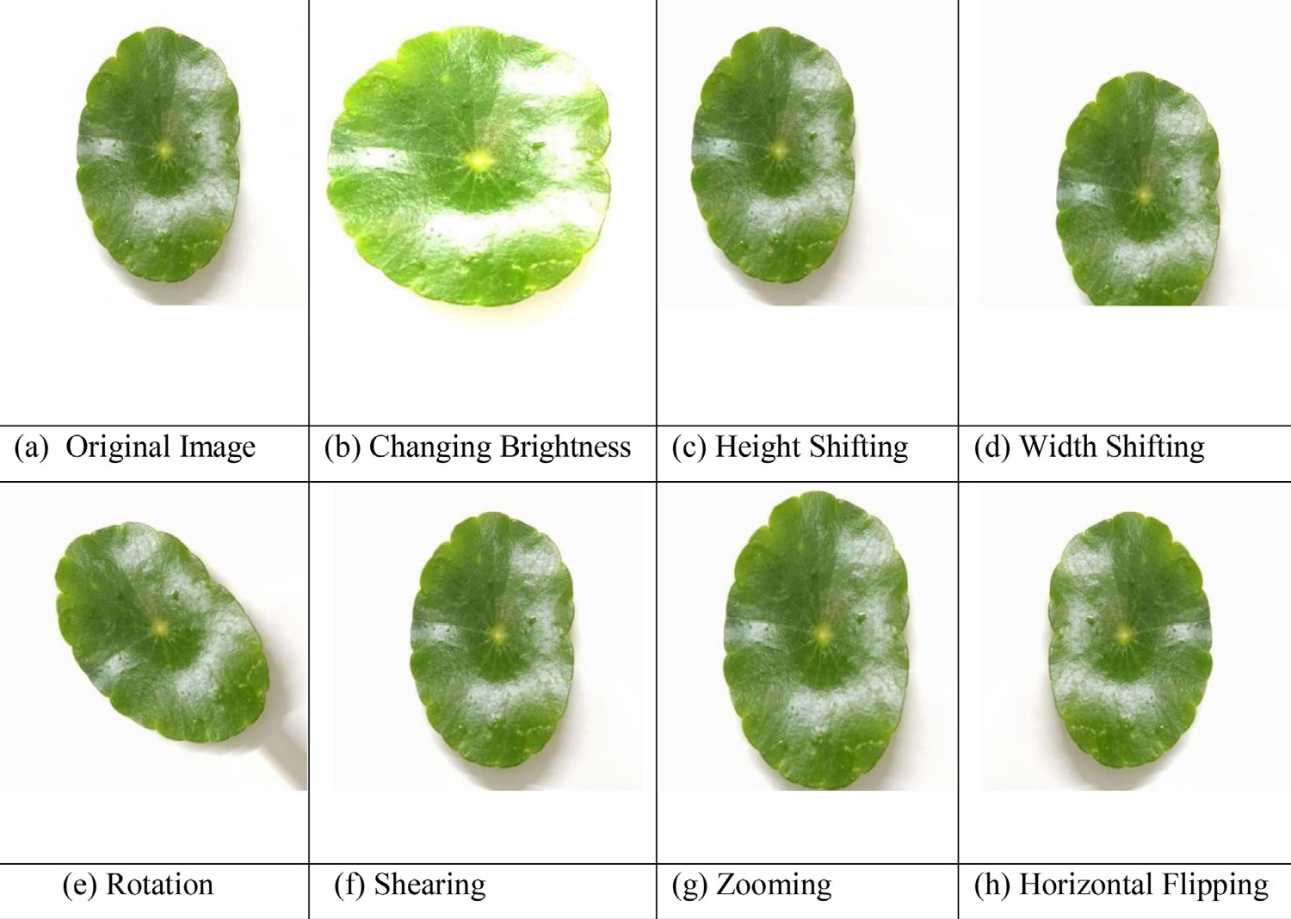
Augmented sample images demonstrating various transformations applied to the original data.

Table 1 presents the class-wise distribution of image samples across the dataset. Fig. 3 illustrates the hierarchical folder structure used to organize the dataset for efficient access and compatibility with machine learning workflows. Fig. 4 visualizes the complete dataset generation workflow, outlining the process from nursery visits and plant identification to image acquisition, augmentation, and final dataset publication. Fig. 5 showcases representative augmented image samples, highlighting the various transformations-such as brightness adjustment, shifting, rotation, shearing, zooming, and flipping-applied to increase dataset variability and support model generalization.

**Table 1:** Statistics of the dataset.

Sl.	Folder name	Original images	Augmented images
1	Arjun Leaf	230	1610
2	Curry Leaf	230	1610
3	Marsh Pennywort Leaf	230	1610
4	Mint Leaf	230	1610
5	Neem Leaf	230	1610
6	Rubble Leaf	230	1610
Total		1380	9660

The complete dataset is publicly available in the Mendeley Data repository [6] for open access and reproducibility in computer vision research on computer vision research.

## 4 Experimental Design, Materials and Methods

### 4.1 Dataset generation workflow

The data collection process began with visits to local nurseries, where medicinal plants were identified and selected based on their morphological characteristics. Once identified, raw images of the plant leaves were captured directly in the nursery environment. To maintain consistency and improve image quality, a controlled studio setup was also utilized. All images were taken using the iPhone 13 Pro, ensuring high-resolution capture with advanced autofocus and stabilization features.

Following the collection, the original dataset was systematically organized, and to enhance the diversity and robustness of the dataset, several data augmentation techniques were applied to each original image. These transformations include: Fig. 5(b) Changing Brightness using histogram equalization to improve contrast and visibility; Fig. 5(c) Height Shifting, where the image is vertically translated; Fig. 5(d) Width Shifting, involving horizontal displacement; Fig. 5(e) Rotation, where images are rotated within a 45-degree range to simulate angular variations; Fig. 5(f) Shearing, which introduces geometric distortion along diagonal axes; Fig. 5(g) Zooming, involving slight scaling in and out; and Fig. 5(h) Horizontal Flipping, which mirrors the image along the vertical axis. These augmentation methods were applied uniformly across all classes to increase sample variability and support the development of deep learning models with improved generalization capabilities. Both the original and augmented images were then compiled and uploaded to the Mendeley Data repository for public access.

### 4.2 Camera specification

Images were captured using the iPhone 13 Pro, which incorporates advanced imaging technologies such as Phase Detection Autofocus (PDAF), focus pixels, and AI-based computational photography to ensure precise and high-speed focusing. The device features a triple-lens camera system comprising a wide lens (f/1.5), an ultra-wide lens (f/1.8), and a telephoto lens (f/2.8). The telephoto lens supports up to 3× optical zoom and 15× digital zoom, enabling flexible image capture from various distances, although digital zoom may introduce slight detail degradation. Optical Image Stabilization (OIS) on the wide and telephoto lenses minimizes blur caused by hand movement or low-light conditions. All images were taken in 4:3 aspect ratio at high resolution, ensuring detailed representation suitable for machine vision tasks and botanical analysis. Table 2 provides detailed specifications of the iPhone 13 Pro camera system used for image acquisition in the dataset.

**Table 2:** Specifications of the iPhone 13 Pro Camera System Used for Dataset Image Acquisition.

Specification	Details
Device Model	iPhone 13 Pro
Camera Setup	Triple-lens system (Wide, Ultra-Wide, Telephoto)
Wide Lens	26 mm, f/1.5 aperture, Optical Image Stabilization (OIS)
Ultra-Wide Lens	13 mm, f/1.8 aperture, 120° field of view
Telephoto Lens	77 mm, f/2.8 aperture, 3× optical zoom, 15× digital zoom
Focus Technology	Phase Detection Autofocus (PDAF), Focus Pixels
Image Stabilization	Sensor-shift OIS (wide), OIS (telephoto)
Image Format	JPEG(.jpg)
Aspect Ratio	4:3
Image Resolution	512 × 512 pixels (cropped and resized from original capture)
Lighting Conditions	Natural daylight in controlled nursery environments
Computational Features	Smart HDR 4, Deep Fusion, Night Mode

## Limitations

Despite offering 11,040 images, the dataset’s original samples were collected from only two nurseries in a single geographic region, limiting environmental diversity. While augmentation increases volume, synthetic transformations may not fully capture real-world variance. Additionally, images were taken under natural lighting, which may introduce slight inconsistencies. No multispectral or texture data were recorded, which could further enhance plant identification tasks. Furthermore, the dataset currently includes only six medicinal plant species and was collected in a single geographic region under relatively controlled backgrounds. These limitations restrict its external validity. A future version (v2) is planned to include more species, images captured across multiple regions and seasons, natural backgrounds, and data collected with different devices to enhance generalizability.

## Ethics Statement

In this article, there is no research involving human or animal subjects conducted by the authors. The datasets used in this article are publicly accessible. Proper citation of these datasets is important when using them.

## Funding

This research did not receive any specific grant from funding agencies in the public, commercial, ornot-for-profit sectors.

## Data Availability

Mendeley DataMedicinal Plant Identification Dataset (Original data).
